# Translation and measurement properties of the pelvic floor distress inventory-short form (PFDI-20) in Iranian reproductive age women

**DOI:** 10.1186/s12905-023-02493-y

**Published:** 2023-06-24

**Authors:** Sepideh Mashayekh-Amiri, Mohammad Asghari Jafarabadi, Fatemeh Rashidi, Mojgan Mirghafourvand

**Affiliations:** 1 Students Research Committee, Midwifery Department, Faculty of Nursing and Midwifery, Tabriz University of Medical sciences, Tabriz, Iran; 2 Cabrini Research, Cabrini Health, Melbourne, VIC 3144 Australia; 3 School of Public Health and Preventative Medicine, Faculty of Medicine, Nursing and Health Sciences, Monash University, Melbourne, VIC 3800 Australia; 4 Road Traffic Injury Research Center, Tabriz University of Medical Sciences, Tabriz, Iran; 5 Students Research Committee, Midwifery Department, Faculty of Nursing and Midwifery, Tabriz University of Medical sciences, Tabriz, Iran; 6 Social Determinants of Health Research Center, Department of Midwifery, Faculty of Nursing and Midwifery, Tabriz University of Medical Sciences, Tabriz, Iran; 7 Menopause Andropause Research Center, Ahvaz Jundishapur, University of Medical Sciences, Ahvaz, Iran

**Keywords:** Pelvic floor dysfunction, PFDI-20, Short-form, Validation, Symptom questionnaire, Reproductive age, Quality of life

## Abstract

**Background:**

Every year, millions of women worldwide suffer in silence from pelvic floor disorders (PFDs) as an annoying health problem. Despite the high prevalence rate and negative effects of PFDs on the quality of life, the validity and reliability of pelvic floor distress inventory-short form (PFDI-20) has not been confirmed for Iranian women of reproductive age. Hence, this study aimed to determine measurement properties of PFDI-20 among women of reproductive age in Tabriz, Iran.

**Methods:**

The current study was cross-sectional research that selected 400 women of reproductive age referring to health centers in Tabriz City, by using cluster random sampling from May 2022 to September 2022. Measurement properties of the Persian version of PFDI-20 were determined and evaluated through five steps, including content and face validity within two quantitative and qualitative parts, structural validity by using exploratory factor analysis (EFA) and confirmatory factor analysis (CFA), and reliability testing through internal consistency, test-retest reliability, and measurement error. Moreover, ceiling and floor effects were investigated.

**Results:**

In this research, CVI (content validity index) and CVR (content validity ratio) of PFDI-20 equaled 0.94 and 0.97, respectively. In addition, the EFA process was applied to 20 items and derived the structure of three factors, which explained 58.15% of the total variance. In CFA phase, values of fit indicators (RMSEA = 0.07, SRMR = 0.07, TLI = 0.97, CFI = 0.99, x2/df = 3.19) confirmed the model validity. To determine reliability, Cronbach’s alpha = 0.84; McDonald’s omega (95% CI) = 0.84 (0.82 to 0.87) and Intraclass Correlation Coefficient (95% CI) = 0.98 (0.97 to 0.99) were obtained. Also, the SEM was 2.64, and the SDC indicating the smallest individual change was 8.91. Regarding the inventory feasibility, the ceiling effect was not observed in total value and subscales, while the floor effect in the total score of PFDI-20 equaled 24.0. The latter rate equaled 45.8, 38.3, and 50.8 for subscales POPDI-6, CRADI-8, and UDI-6, respectively.

**Conclusions:**

Persian version of PFDI-20 is a valid and reliable scale used to evaluate PFDs in Iranian women of reproductive age. Healthcare professionals can use this scale to screen PFDs, and researchers can consider it a reliable tool for their studies.

## Background

As the central core of the body, Pelvic Floor (PF) consists of bony, ligamentous, and muscular structures that cover the lower part of the pelvic cavity [1]. Pelvic floor muscles (PFM) consist of coccygeus and Levator ani (pubococcygeus, puborectalis, and iliococcygeus), which together with ligaments and other connective tissue serve as a chain to support pelvic organs, filling and emptying bladder and intestines, control sphincter, and ensure the reproduction and sexual functions. Therefore, any damage to PFM and pelvic fascia leads to pelvic floor disorders (PFDs) [2].

PFDs include a wide range of disorders, including urinary incontinence (stress, urge, and mixed UI), fecal incontinence (FI), Pelvic organ prolapse (POP), sexual dysfunction, diastasis recti abdominis, pelvic girdle pain, and chronic pain syndromes [3, 4].Overall, PFDs affect 23–49% of women [4, 5]; hence, anticipations indicate that this rate will increase to 43.8 million cases in developing and developed countries by 2050. In this case, stress urinary incontinence (SUI) will be the most prevalent case with a rate of 17% [6]. Most PFDs occur in the reproductive age of women, especially in the last months of pregnancy and after childbirth. This trend become ascending in line with the aging process [7].

PFDs are multifactorial disorders with unknown causes [8]. According to available examinations, some factors, such as age [9], parity [10], mode of delivery [11], body mass index (BMI) [12], pelvic surgery [13], spinal disorders [14], genetics [15], and chronic cough [16] may cause such disorders [17]. In this case, pregnancy and childbirth are the most critical risk factors that weaken the pelvic floor muscles because of physiological and hormonal changes [18, 19].

As a disabling and annoying problem, PFDs dramatically affect the physical, psychological, social, and functional aspects of women putting them at risk of many problems, including sexual problems [20], social issues [21], psychological and functional disorders [22], isolation and lack of self-confidence [23], inability to do their religious tasks, sense of guilt, sleep disorders [24], and depression [25]. Affected women feel impure or embarrassed due to urine or fecal incontinence during the day or sexual intercourse, which negatively affects their quality of life (QoL) [26].

However, affected women consider these symptoms normal consequences of childbirth or aging, and do not go to medical centers postponing the diagnosis and treatment of PFDs [27]. Hence, early diagnosis of these symptoms depends on some factors, such as access and assessment by health care providers. Therefore, healthcare providers are responsible to examine, screen, and evaluate these disorders accurately [28, 29]. Because the evaluation of PFDs is a mental assessment that requires examining women’s perception of symptoms [30], questionnaires are the best methods used to screen women with PFDs. The reason is that questionnaires are low-invasive, reproducible, and inexpensive, evaluate symptoms and QoL of women with PFDs, and can effectively treat these disorders [31].

Now, the International Continence Society (ICI) frequently recommends some tools and measures to examine PFDs. International Consultation on Incontinence Questionnaire (ICIQ) is the first version of a measure that examines bladder disorders while developing some options concerning POP and colorectal disorders. This tool is mostly used to assess a range of bladder diseases [32]. The pelvic Floor Impact Questionnaire (PFIQ-7) is another recommended tool that measures the impact of PFDs on health-related quality of life (HRQoL) [33]. However, ICI has widely recommended Pelvic Floor Distress Inventory-20 (PFDI-20) as a grade-A tool for PFDs. Patient-reported outcome measures (PROMs) are the most reliable criteria for examining the presence, severity and impact of pelvic floor disorders in clinical practice [31]. The Consensus-Based Standards for the Selection of Health Measurement Instruments (COSMIN) produced updated guidelines for evaluating PROMs from different domains of knowledge [34]. PFDI-20 is a PROMs, has been translated into several languages and confirmed worldwide. PFDI-20 is the short version of PFDI-46 that was designed and introduced by Barber et al. (2005) in the US. The PFDI-20 assesses PFDs through 20 items in three factors of Urinary Distress Inventory 6 (UDI-6), Colorectal-Anal Distress Inventory-8 (CRADI-8), and Pelvic Organ Prolapse Distress Inventory 6 (POPDI-6) [35].

Regarding the high prevalence of PFDs in women of reproductive ages and no demand for treatment, the role of PFDs screening by health care providers becomes highlighted more than before. However, the validity and reliability of PFDI-20 have not been examined for Iranian women of reproductive age. Hence, the present study aimed to determine the measurement properties of PFDI-20 among Iranian women of reproductive age.

## Methods

### Participants and study design

This was a cross-sectional study conducted to determine measurement properties of the Persian version of PFDI-20 on 400 women of reproductive age referring to healthcare centers in Tabriz, Iran. After obtaining the license and following all ethical principles, this study was conducted from May 2022 to September 2022.

### Translation process

After getting the tool designers’ permission (Barber et al.) [35], the translation process was started following the WHO’s guidelines, Eortc Quality of Life Group Translation Procedure Guidelines, and expert panel review [36]. The translation process was done by using two Forward-Backward (FB) and Dual Panel (DP) methods within four phases: (1) Forward- Translation, (2) Backward-Translation, (3) Pre-testing and cognitive interviewing, and (4) Final version.

Forward translation requires two separate translations from the source language (English) to the destination language (Persian). For this purpose, the main English version of the tool was translated into Persian separately by two native persons who had mastery of English and were skilled in designing PFDs measures (emphasizing conceptual, not literal translation and using a language that is comprehensible for the majority of audiences). These two translators then investigated differences between translated versions (reconciled translation) and presented a single version after removing the conflicts [37].

Backward translation was used to ensure that the Persian translation matched the original version. The questionnaire was again translated into English by two native translators who were not involved in the translation process of the original version and did not see the original version. The final report at the end of this step included two Forward translations from English to Persian, reconciled translation, and two backward translations from Persian into English. The expert panel then added comments about translations. Ultimately, a pilot study must be done in this group before using the tool in the target population. To do this, questionnaires were distributed among 10 eligible women, and their comments were taken to change the Persian version regarding comprehensibility, grammar, writing style, and easy completion process. The final version was prepared in the last step [37].

### Population and sample

The sample size must be calculated to do the factor analysis process. Some researchers consider a sample size of 200–300 sufficient. Costello et al. introduce the subject-to-item ratio as the best method for determining sample size. They believe that 10–20 participants must be taken for each item of the tool [38]. Therefore, this study chose 10 participants for each item, applied Design effect = 2 due to cluster sampling, and selected 400 women of reproductive age referring to healthcare centers in Tabriz, by using the cluster sampling method.

In the sampling process, one-fourth of centers were selected randomly (www.random.org website) then a list of women (names and phone numbers) of women who were in the reproductive age (older than 15 years) were extracted from the SIB system (integrated health system). All women were invited to participate in the study, regardless of having diagnosis of PFD or not. The number of women chosen from each center was proportionally calculated concerning and they were chosen at random using the same website. Then, the researcher called women using their phone number, briefly explained the aims for the study and how it would be carried out, and extended an invitation to participate. Women were asked to come to the health center on a specific time to complete the questionnaires.

After they attended in the health center, women were examined regarding inclusion and exclusion criteria then they signed consent letters, and the researcher asked them about urine analysis (U/A) test in the past three days. The U/A was prescribed for those who did not have this test in the past three days. The test results were examined and women with white blood cell (WBC) > 3 were excluded from the study.

Inclusion criteria comprised women older than 15, having sex, monogamous couple, and no pregnancy during the research period. Women with urinary tract infections, gynecologic surgery, including restorative and cosmetic surgeries, and sexually transmitted diseases (STDs) were excluded from the study. Finally, the socio-demographic and obstetric characteristics questionnaire and PDFI-20 were completed through interview with participants.

### Measurement instruments

1. *Socio-demographic and obstetric history checklist*

This questionnaire comprised some information, including age, weight, height, BMI, Gravidity, Parity, education level, job, mode of delivery, and family history of PFDs.

2. *Pelvic floor distress inventory-short form-20 (PFDI-20)*

Barber et al. (2005) designed this tool in the USA. This tool comprises 20 items that evaluate the PFDs within three factors entitled Pelvic Organ Prolapse Distress Inventory 6 (POPDI-6), Colorectal-Anal Distress Inventory-8 (CRADI-8), and Urinary Distress Inventory 6 (UDI-6). Each item is scored between 0 and 4. If the answer is No, the score equals 0, and if the score equals 1–4 then items are scored based on symptoms’ intensity (0 = not present, 1 = not at all, 2 = somewhat, 3 = moderately, and 4 = quite a bit). Scores on the scale are measured by calculating the mean score of each scope and multiplying it by 25. The maximum score of each scale varies between 0 (least distress) and 100 (greatest distress). The total score (0-300) is calculated by summing up the scores of three scales. The higher the score, the more the PFDs will be [35]. The measurement properties of the PFDI-20 reported by Arruda et al. showed the one-dimensional structure with Cronbach’s alpha = 0.929, and the distress caused by the presence of PFD symptoms can be classified as mild (1 to 15 points), moderate (16 to 34 points), and severe (35 to 40 points) [39].

### Statistical analysis

Data analysis was done through SPSS Statistics 16 (IBM Corp, Armonk, NY, USA), STATA 14 (Statcorp, college station, Texas, USA), and R software 4.2 (Psych package). This study used descriptive and analytical indicators, including Mean (SD) for quantitative variables and used frequency (percent) for qualitative variables to describe socio-demographic characteristics and obstetric background.

### Validity (content validity, face validity, construct validity)

#### Content validity

Content validity points to the extent to which a tool contains suitable items for a considered construct. Two qualitative (based on the comments of the expert panel and target group) and quantitative (based on measurement of CVR (content validity ratio) and CVI (content validity index)) methods were used to assess content validity [40].

### Qualitative content validity

The qualitative content validity of the tool is determined based on the expert panel’s comments. Content experts are professionals who are skilled in designing the tool and context of PFDs, while lay experts are target populations and research subjects [41]. To determine qualitative content validity, the researcher asked about the opinions of ten experts (reproductive health, midwifery, and nursing education experts) and 10 women from the target group. They were asked to present their ideas about the general structure of the questionnaire, items’ content, removal or adding items, Persian grammar, use of proper words and grammar, and accurate scoring. The questionnaire was then changed based on the experts’ feedback [40].

### Quantitative content validity

To examine CVR, the expert panel’s members were asked to review items accurately the leave and evaluate their comments about the items based on a 3-point Likert scale (necessary, useful but unnecessary, unnecessary). Finally, CVR was measured based on this equation: CVR= (“Ne”-“N”/2)/(“N”/2) where “Ne” indicates the number of experts that consider the case necessary, and “N” represents the total number of experts. Accordingly, the CVR > 0.62 confirmed the necessity of items based on the Lawshe table in the present study that the expert panel had 10 members [42].

CVI was measured based on the comments of the expert panel (10 members) and the Waltz and Basel index in the next step [43] through which, experts investigated three criteria of relevance, clarity, and simplicity for each item based on the 4-point Likert scale. CVI was measured based on the following formula, and its score > 0.79 was confirmed [44].

CVI = the number of specialists who gave 3 and 4 for the items/N.

### Face validity

Face validity was examined based on two qualitative (regarding the comments of the expert panel and target group) and quantitative (measuring Impact score) approaches [45]. Researchers find the face validity of a measure based on the comments of target groups and experts. Face validity indeed indicates the apparent attraction of a tool that may influence the confirm ability of the tool by participants [45].

### Qualitative face validity

In the qualitative method, the researcher interviewed 10 eligible women referring to healthcare centers in Tabriz. The following points were considered in interviews: the difficulty level of items, optimal fit, the relationship between items and the main purpose of the tool, ambiguity, and misinterpretation of items. Although content experts play a vital role in content validity, members of the target society revised the tool since revision is another important component of content validation [46].

### Quantitative face validity

Quantitative face validity was evaluated based on the comments of 10 end users of the questionnaire about items based on a 5-point Likert Scale (very important, important, relatively important, slightly important, and unimportant). The impact was then used to measure the percent of women that gave scores of 4 or 5 to the importance of items (frequency), mean score of item’s importance (importance), and impact score based on the following formula: Impact Score = Frequency (%) × Importance. The items would be kept if the Impact score ≥ 1.5, removed, otherwise [47].

### Structural validity

Structural validity was evaluated by using exploratory factor analysis (EFA)based on two Kaiser-Meyer Olkin (KMO) and Bartlett’s test of Sphericity measures through core components analysis with varimax rotation (direct oblimin) [48]. It is worth noting factor load was considered greater than 0.3. In the case of EFA and component analysis, the KMO measure is calculated for sample adequacy, which equals 0.8 and greater [49]. Moreover, Bartlett’s test of Sphericity is done to assess the appropriateness of factor analysis. The significance of this test indicates a confirmed matrix of correlation between these items and appropriate factor analysis [50]. On other hand, some indicators were used to examine model fit in the confirmatory factor analysis (CFA) phase [51, 52]:

Root mean score error of approximation (RMSEA < 0.08), standardized root mean square residual (SRMR < 0.10), normed Chi^2^ (x^2^ / df) < 5, comparative fit indices including comparative fit index (CFI > 0.90) and Tucker-Lewis Index (TLI) > 0.90.

### Feasibility

Finally, one of the important components of this validation after factor analysis is identifying floor and ceiling effects (F/C), which indicates the power of a questionnaire in distinguishing respondents at the end of the scale. F/C effects are defined as the ratio of respondents that obtain the highest (ceiling) or lowest (floor) score in each field. The F/C effect measures the sensitivity and coverage of a questionnaire at two extremes of the scale [53].

### Reliability

The concept of reliability means achieving similar results in frequent measurements using a single tool. The present study examined the reliability of the inventory using internal consistency and calculating Cronbach’s alpha coefficient and McDonald’s Omega Coefficient, and intra-class correlation coefficient (ICC) (2-way mixed-effects model with single rater/measurement type) through the test-retest method [54, 55]. Internal consistency of the tool was measured for the whole questionnaire using Cronbach’s alpha coefficient and McDonald’s Omega Coefficient for each scope. The optimal rate of internal consistency was greater than 0.7. Moreover, 30 women of reproductive age referring to the healthcare centers of Tabriz filled out the questionnaires within two-week intervals to determine ICC. The correlation between scores obtained from the two assessments was determined with ICC and confidence intervals; the ICC values greater than 0.6 indicated desired consistency [56]. Measurement error can be indicated as the standard error of measurement (SEM) and the smallest detectable change (SDC) or minimal detectable change (MDC). The SEM describes the SD of repeated measures in one patient and was calculated using the square root of the error variance [34]. The SDC or MDC describes the smallest individual change that a patient needs to show on the scale to ensure that the observed change is real [34].

### Ethical consideration

The present study was approved by the ethics committee of Tabriz University of Medical Sciences (ref: IR.TBZMED.REC.1400.1073). Researchers got permission from the designers of this tool (Barber et al.) before starting the study then explained the research process to all participants and asked them to sign a consent letter. The author ensured participants about the confidentiality and allowed them to leave the research process anytime they wanted to exit.

## Results

### Baseline characteristics

Generally, 400 women of reproductive age entered the study randomly from May 2022 to September 2022. The mean (SD) of participants’ age equaled 34.4 (7.2), ranging from 16 to 49, and more than three-fourths of them (95.8%) did not report family background of PFDs (Table 1). The mean (SD) of whole scale equaled 27.3 (31.1), while equaled 8.6 (11.8), 9.9 (12.2), and 8.8 (13.4) for three extracted factors of POPDI-6, CARDI-8, and UDI-6, respectively.

**Table 1:** Baseline characteristics of participants for factor analysis of PFDI-20 (n = 400)

Characteristics	Mean	SD
**Age** (Year)	34.4	7.2
**BMI** (kg/m^2^)	26.9	4.1
	**Number**	**Percent**
**Educational level**		
High school or below	286	71.5
Diploma and university	114	28.5
**Gravidity**		
Two and lower	299	74.8
Three and more	101	25.3
**Parity**		
Two and lower	352	88.0
Three and more	48	12.0
**Occupation**		
Housewife	329	82.3
Employee	71	17.8
**Income**		
Not at all sufficient	74	18.5
Relatively sufficient	229	57.0
Completely sufficient	98	24.5
**Type of delivery**		
NVD with Episiotomy	125	32.9
NVD without Episiotomy	13	3.4
C/S	210	55.3
Both	32	8.4
**Family history of PFDs**		
Yes	17	4.2
No	383	95.8

SD Standard deviation, BMI body mass index, NVD normal vaginal delivery, C/S cesarean section, PFDs pelvic floor dysfunctions

### Content and face validity

In the case of content validity assessment, all items had the minimum acceptable CVI, and CVR values equaled 0.94 and 0.97, respectively. In the case of face validity, all items were fit and free of any ambiguity and difficulty and received a minimum score of 1.5 (Table 2).

**Table 2:** The results for the content and face validity of the Iranian version of PFDI-20 (n = 10)

Item	CVI	CVR	Impact score
1. POPDI1	1.00	1.00	3.46
2. POPDI2	0.91	1.00	3.33
3. POPDI3	0.87	1.00	3.20
4. POPDI4	1.00	1.00	3.06
5. POPDI5	1.00	1.00	3.46
6. POPDI6	1.00	1.00	3.86
7. CRADI1	0.83	1.00	3.46
8. CRADI2	1.00	1.00	3.86
9. CRADI3	0.54	0.75	3.60
10. CRADI4	1.00	1.00	3.86
11. CRADI5	1.00	1.00	3.86
12. CRADI6	1.00	1.00	4.00
13. CRADI7	1.00	1.00	3.46
14. CRADI8	1.00	1.00	3.86
15. UDI1	1.00	1.00	4.00
16. UDI2	0.87	0.87	3.60
17. UDI3	095	0.87	4.00
18. UDI4	1.00	1.00	4.00
19. UDI5	1.00	1.00	4.00
20. UDI6	0.87	0.87	4.00

CVI Content Validity Index; CVR Content Validity Ratio; POPDI-6 Pelvic Organ prolapse Distress Inventory 6; CRADI-8 Colorectal-Anal distress Inventory-8; UDI-6 Urinary distress Inventory 6

### Structural validity

**Fig. 1:**
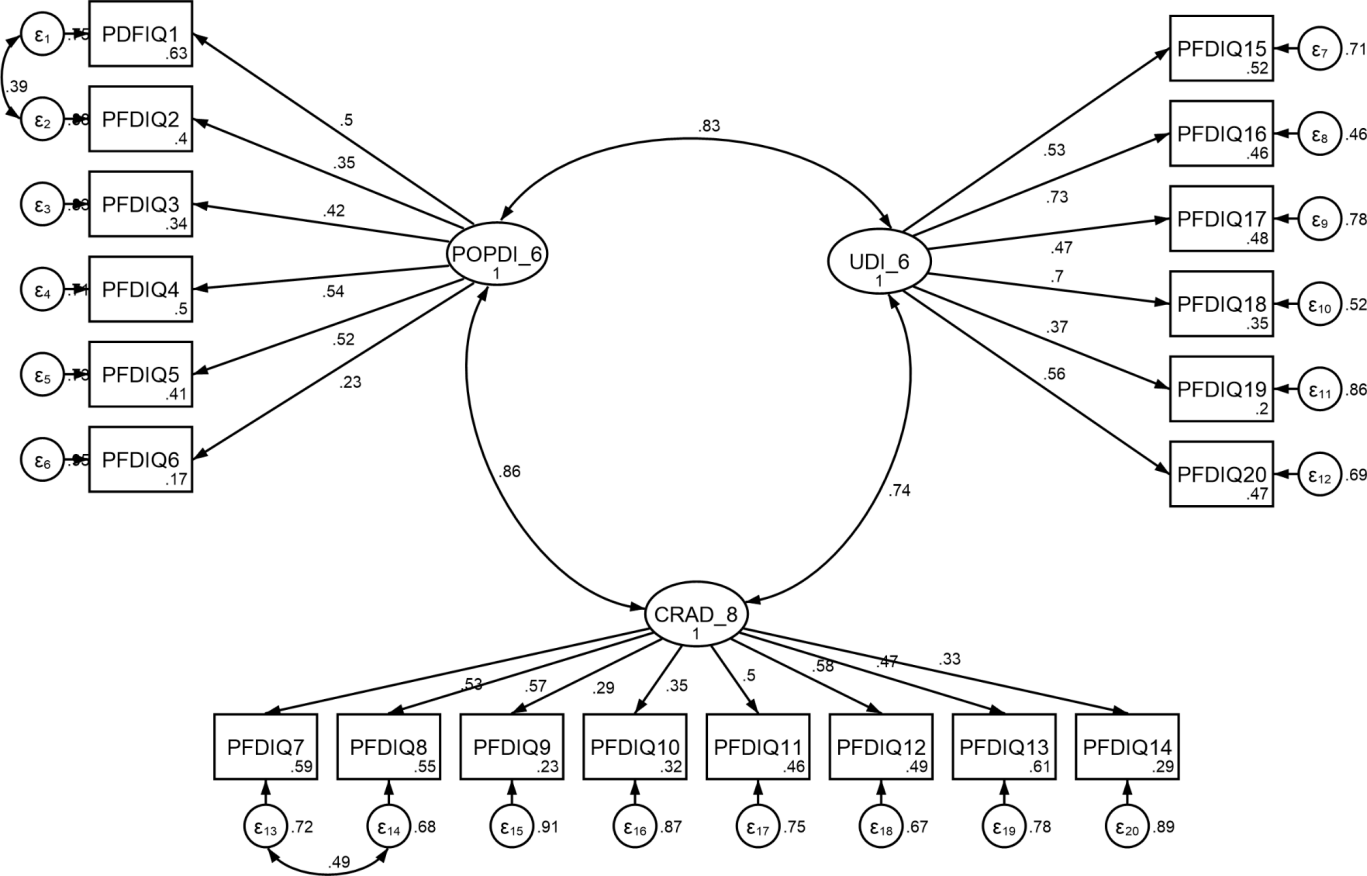
Factor structure model of the PFDI-20 based on CFA (All factor loadings are significant at p < 0.001) POPDI-6 Pelvic Organ prolapse Distress Inventory 6; CRADI-8 Colorectal-Anal distress Inventory-8; UDI-6 Urinary distress Inventory 6

Structural validity was evaluated through EFA and CFA on 400 women of reproductive age (conducting EFA and CFA on same sample). Kaiser-Meyer-Olkin (KMO) value equaled 0.726. The KMO > 0.7 confirmed the significance of Bartlett’s test and model adequacy (P ≤ 0.001). Moreover, the three-factor structure in the EFA process obtained a total variance of 58.15% (Table 3). The first factor was POPDI-6, which comprised 6 items that explained 18.25% of the total variance. It should be mentioned that item 6 of factor POPDI-6 has a factor loading of less than 0.3, but according to the opinion of the research team, this item had a high importance and weight and we could not remove it, but it should be noted that in the CFA, this item was significant (p < 0.001), so it was not removed. CRADI-8 was the second factor that covered 8 items that explained 17.10% of the total variance. Finally, the third factor included UDI-6 comprised 6 items that explained 22.80% of the whole variance. Also, the results showed a significant correlation between the items PDFIQ1 and PDFIQ2 (r = 0.39, P < 0.05), as well as the items PDFIQ7 and PDFIQ8 (r = 0.49, P < 0.05) (Fig. 1).

**Table 3:** Result of Facture analysis of the PFDI-20 scale based on EFA (n = 400)

Scale item		Factors	
	1	2	3
**Factor 1: POPDI-6**			
1. Usually experience pressure in the lower abdomen?	0.673		
2. Usually experience heaviness or dullness in the pelvic area?	0.575		
3. Usually have a bulge or something falling out that you can see or feel in your vaginal area?	0.419		
4. Ever have to push on the vagina or around the rectum to have or complete a bowel movement?	0.438		
5. Usually experience a feeling of incomplete bladder emptying?	0.420		
6. Ever have to push up on a bulge in the vaginal area with your fingers to start or complete urination?	0.193		
**Factor 2: CRADI-8**			
7. Feel you need to strain too hard to have a bowel movement?		0.687	
8. Feel you have not completely emptied your bowels at the end of a bowel movement?		0.719	
9. Usually lose stool beyond your control if your stool is well formed?		0.269	
10. Usually lose stool beyond your control if your stool is loose?		0.302	
11. Usually lose gas from the rectum beyond your control?		0.446	
12. Usually have pain when you pass your stool?		0.593	
13. Experience a strong sense of urgency and have to rush to the bathroom to have a bowel movement?		0.375	
14. Does part of your bowel ever pass through the rectum and bulge outside during or after a bowel movement?		0.301	
**Factor 3: UDI-6**			
15. Usually experience frequent urination?			0.550
16. Usually experience urine leakage associated with a feeling of urgency, that is, a strong sensation of needing to go to the bathroom?			0.729
17. Usually experience urine leakage related to coughing, sneezing or laughing?			0.487
18. Usually experience small amounts of urine leakage (that is, drops)?			0.724
19. Usually experience difficulty emptying your bladder?			0.354
20. Usually experience pain or discomfort in the lower abdomen or genital region?			0.507
**% of variance observed**	18.25	17.10	22.80
**Total score**	58.15		

PFDI-20 Pelvic Floor Disability Index; POPDI-6 Pelvic Organ prolapse Distress Inventory 6; CRADI-8 Colorectal-Anal distress Inventory-8; UDI-6 Urinary distress Inventory 6

In the CFA phase, three factors were obtained and then used in EFA by CFA. According to results (RMSEA = 0.07, SRMR = 0.07, TLI = 0.97, CFI = 0.99, x_2_/df (normed chi-square) = 3.19), this model had an optimal fit. Hence, factor structure could be confirmed (Table 4).

**Table 4:** The model fit indicators of the PFDI-20 (n = 400)

Goodness of fit indices	CFA	Acceptable value
**χ2**	526.954	
**Df**	165	
*x*^2^/df	3.194	< 5
**P-value**	< 0.001	0.05>
**CFI**	0.999	> 0.90
**TLI**	0.968	> 0.90
**SRMR**	0.066	< 0.10
**RMSEA (90% CI)**	0.074 (0.067–0.081)	< 0.08

χ2 chi-square; df degrees of freedom; χ2/df normed chi-square; CFI Comparative Fit Index; TLI Tucker–Lewis index; SRMR Standardized root mean squared residual; RMSEA root mean square error of approximation

### Feasibility

The ceiling effect was not observed in total value and subscales, while the floor effect in total score equaled 24.0%, while this effect equaled 45.8, 38.3, and 50.8% for POPDI-6, CRADI-8, and UDI-6 subscales, respectively.

### Reliability

Cronbach’s alpha coefficient and McDonald’s omega (95% CI), which equaled 0.84 and 0.84 (0.82–0.87), respectively indicating optimal internal consistency of the questionnaire. In the test-retest method, ICC (95% CI) equaled 0.98 (0.97–0.99). SEM is a measure that helps us determine the precision and reliability of a measurement. In this case, the SEM was found to be 2.64. This means that if we were to repeat the measurement multiple times, we would expect the values to fall within a range of ± 2.64 units around the true score. Additionally, SDC indicates the minimum amount of change that can be reliably detected by the measurement tool. In this context, the SDC was determined to be 8.91 units. This means that any individual change in the measured quantity that is smaller than 8.91 units may not be distinguishable from the measurement error and could be considered insignificant (Table 5).

**Table 5:** Stability Coefficients and Interclass Correlation Coefficient of the PFDI-20

Factors	Cronbach’s α coefficient	McDonald’s omega (95% CI)	ICC (95% CI)	SEM	SDC
POPDI-6	0.63	0.64 (0.58, 0.69)	0.95 (0.90, 0.98)	1.30	3.60
CRADI-8	0.70	0.73 (0.68, 0.77)	0.96 (0.92,0.98)	1.70	4.71
UDI-6	0.72	0.73 (0.70, 0.77)	0.99 (0.97,0.99)	1.48	4.10
PFDI-20 (Total)	0.84	0.84 (0.82, 0.87)	0.98 (0.97,0.99)	2.64	8.91

ICC intra class correlation coefficient; CI confidence interval; POPDI-6 Pelvic Organ prolapse Distress Inventory 6; CRADI-8 Colorectal-Anal distress Inventory-8; UDI-6 Urinary distress Inventory 6; PFDI-20 Pelvic Floor Disability Index; SEM Standard error of the measurement; SDC smallest detectable change

## Discussion

In this study, we evaluated the measurement psychometric properties of PFDI-20 among Iranian women of reproductive age according to the COSMIN. Based on the results of this study, the Persian version of this questionnaire is a reliable tool for evaluating PFDs among women of reproductive age in the Iranian community. Generally, PFDs affect the lives of many women worldwide because they do not see it as an abnormal case and are not aware of available treatment options. Hence, this negligence has reduced the quality of life of women, affected their role in family health, and led to many complications. Hence, PFDs must be screened to diagnose them timely by measuring them through valid and reliable tools [57].

Many patient-reported outcome measures (PROMs) have been created for daily use and clinical research over three recent decades. However, a high number of published PROMs confused urogynecologists who faced a challenge in selecting the best and most comprehensive tool [58]. In this regard, five specific chapters exist to cover PROMs in gynecological urology and urogynecology textbooks. These chapters consist of 105 PFDs questionnaires, including HRQL, symptom bother, urgency-specific measures, screener satisfaction, goal assessment tools, bowel dysfunction, sexual dysfunction, POP, and Electronic Personal Assessment Questionnaire Pelvic Floor [59].

In 2017, the 6th ICI advised using 12 symptoms bother measures and 33 HRQL measures for lower urinary tract symptoms (LUTS), 13 urinary urgency measures, 10 measures for FI, other bowel symptoms, 8 patient satisfaction questionnaires, 17 screening tools, and 5 sexual health and QoL measures [59]. In women’s health, PFDI-20 is a PROM that is mainly used in clinical practice and research to evaluate the bothersome caused by PFDs. This PROM obtained a grade of A from International Consultation on Incontinence (ICI) in clinical practice [35].

According to the COSMIN checklist, the functionality of the tool must be an assessment by using EFA when a tool does not have an integrated factor structure, few studies have found different factors or a certain method has not been used for structural analysis [34]. CFA is used in the next step to confirm results adequacy. Therefore, it is necessary to evaluate the measurement properties of PFDI-20, especially structural validity to test its dimensions and internal consistency, as well as whether this PROM is suitable for use in clinical practice and academic research.

According to the EFA process in this study, 3 factors (POPDI-6, CRADI-8, and UDI-6) were extracted for 20 items of the questionnaire, which explained around 58.15% of the variance. Although psychometric features of PFDI-20 have been examined in several languages in the world [60], factor structure was only found in two studies. The first case was the Chinese version by Ma et al., that extracted five factors (anal and colorectal distress (factor 1); direct POP feelings and symptoms of irritation or obstruction of the lower urinary tract (factor 2); various types of urinary incontinence (UI) (factor 3); external force to defecate (factor 4); and symptoms of rectocele (factor 5)), which explained 69.55% of the variance with Cronbach’s alpha = 0.88. In this version, items of each subscale in different factors were integrated, but EFA was not done [61]. The second case was the Brazilian version reported just for one extracted factor (43.74%) and performed EFA and CFA [39]. Furthermore, in the study of Barber et al., [33], three factors were extracted, which are in line with the present study. In contrast to the study by Barber et al. [33], in the present study, the short version of the PFDI (PFDI-20 versus PFDI-46) was used. It should be noted that psychometric tests of PFDI-20 were done for Iranian menopausal women in 2017, while EFA and CFA were not done in this version. This version was published as an Abstract without full information about psychometric tests [62]. Moreover, the KMO value and significant Bartlett’s test confirmed model adequacy regarding the confirmation of the tool’s validity in this study.

In line with Swedish and Dutch studies [63, 64], no ceiling effect was observed in the present study for total scores or subscales of these measures. However, floor effects were found in PFDI-20 and its three factors. Hence, it is recommended to interpret PFDI-20 based on the total score and subscale score. These findings confirm the Dutch study, which found a similar floor effect [64].

Cronbach’s alpha coefficient of the whole questionnaire and its range for extracted subscales equaled 0.84 and 0.63–0.72, respectively, that indicating satisfying internal consistency that was matched with values reported in Brazilian [65], Norwegian [66], Chinese [61], and Finnish [67] versions. In this study, ICC was used to determine test-retest reliability; this rate equaled 0.99, which was greater than the value reported in the original version (0.86). Moreover, the obtained results were in line with the Chinese versions [61] but greater than the values reported in the Swedish [63] and Finnish [67] versions.

Despite the high prevalence of PFDs, there are few studies and antecedents about the most optimal management solution under such circumstances. On the other hand, the lack of referrals by Iranian women for treatment due to shame, the use of this tool is very important in the clinical practice, in order to screen and identify PFDs. Therefore, a special tool for the evaluation of PFDs symptoms in women of reproductive age helps health care providers to diagnose and treat this disorder in its early stages.

### Strength and limitation

The strength of this study is the assessment of measurement properties of PFDI-20 among Iranian women of reproductive age for the first time by integrating dual panel and FB method for the translation process to overcome the FB-method constraint and compare it with other versions. On the other hand, this study faced some limitations: first, not calculating criterion validity due to the lack of a gold standard for PFDs symptoms; second, considering a set of identical data for CFA and EFA, and potential bias caused by the willingness to give optimal social answers with self-report measures. Other limitations include lack of hypothesis testing for construct validity (comparison with another instrument or between groups), not evaluating of cross cultural validity and local sample due to lack of generalizability to the whole country.

## Conclusion

Persian version of PFDI-20 is a valid and reliable scale used to evaluate PFDs in Iranian women of reproductive age. Healthcare professionals can use this scale to screen PFDs, and researchers can consider it a reliable tool for their studies. It seems that this tool can be used for screening and early diagnosis of PFDs among Iranian women of reproductive age to improve their quality of life and reduce the healthcare costs caused by complications of this situation.

## Data Availability

The datasets generated and/or analyzed during the current study are not publicly available due to the limitations of ethical approval involving the patient data and anonymity, but are available from the corresponding author upon reasonable requests.

## Abbreviations

PFDs: Pelvic floor disorders
PFDI-20: Pelvic floor distress inventory-short form
POPDI-6: Pelvic Organ prolapse Distress Inventory 6
CRADI-8: Colorectal-Anal distress Inventory-8
UDI-6: Urinary distress Inventory 6
QOL: Quality of life
POP: Pelvic organ prolapse
PROMs: Patient report outcomes
EFA: Exploratory factor analysis
CFA: Confirmatory factor analysis
ICC: Intra-class Correlation Coefficient
SD: Standard deviation
CVI: Content validity index
CVR: Content validity ratio
df: Degree of freedom
KMO: Kaiser-Meyer-Olkin
RMSEA: Root mean squared error of approximation
CFI: Comparative fit index
TLI: Tucker–Lewis index
